# Time series analysis of environmental factors and their association with emergency visits for asthma exacerbations in Petaling Jaya, Malaysia

**DOI:** 10.7189/jogh.16.04249

**Published:** 2026-08-07

**Authors:** Norita Hussein, Wee Cheah, Chun Lin, Chng Saun Fong, Nasrin Aghamohammadi, Nik Sherina Hanafi, Yong Kek Pang, Mohmmad Salleh Yahya, Ahmad Tajuddin M Nor, Hilary Pinnock, Harry Campbell, Ee Ming Khoo

**Affiliations:** 1 Department of Primary Care Medicine, Faculty of Medicine, Universiti Malaya, Malaysia; 2 Institute of Ocean and Earth Sciences, Universiti Malaya, Malaysia; 3 National Institute for Health Research Global Health Research Unit on Respiratory Health, Usher Institute, University of Edinburgh, UK; 4 Institute for Advanced Studies, Universiti Malaya, Malaysia; 5 Department of Social and Preventive Medicine, Faculty of Medicine, Universiti Malaya, Malaysia; 6 Curtin University Sustainability Policy Institute, School of Design and the Built Environment, Curtin University, Perth, Australia; 7 Department of Medicine, Faculty of Medicine, Universiti Malaya, Malaysia; 8 Trauma and Emergency Unit, Universiti Malaya Medical Centre, Kuala Lumpur, Malaysia; 9 Department of Emergency and Trauma, Hospital Tengku Ampuan Rahimah, Klang, Malaysia

**Keywords:** non-conventional lipid parameters, hs-CRP, hypertension, cohort, CHARLS

## Abstract

**Background:**

The burden of respiratory conditions, especially asthma, is likely to increase with urbanisation and pollution unless effectively managed. Petaling Jaya, a metropolitan area, faced heightened risk due to worsening outdoor air pollution, which can trigger asthma exacerbations. We aimed to examine the relationships between environmental factors and emergency visits for asthma exacerbations.

**Methods:**

We used retrospective data over four years (1 January 2014 to 13 April 2017) comprising clinical records from a main hospital in Klang Valley and environmental data (air pollutants and meteorological data) obtained from nearby stations. We used generalised additive models to assess the associations between environmental factors and asthma emergency visits. We used distributed lag nonlinear models to address the delayed effects and nonlinearity of particulate matter at 10 μm (PM_10_) exposure on these visits.

**Results:**

Asthma exacerbations accounted for 2.3% of total emergency visits. Daily mean (x̄) PM_10_ level exceeding Malaysian Ambient Air Quality Standard (MAAQS) threshold was associated with a 21.3% (95% confidence interval (CI) = 2.2, 44.0) increase when levels reached 200 μg/m^3^ and 34.0% (95% CI = 4.7, 71.7) increase when levels reached 300 μg/m^3^ in asthma emergency visits. The lag effect was associated with PM_10_ exposure levels, peaking on days two and three at 100 μg/m^3^ and on the same day at 200 μg/m^3^ and 250 μg/m^3^. The cumulative exposure-response curve from lag 0–7 days shows that the increased relative risk of asthma emergency visits began at PM_10_ levels of 150 μg/m^3^.

**Conclusions:**

We provide preliminary insights into the respiratory health burden in the Klang Valley. The associations observed between PM_10_ and asthma emergency visits are exploratory. Our findings suggest that the risk of visits may increase when PM_10_ levels near or above current MAAQS thresholds, highlighting the potential benefit of aligning local air quality standards with stricter international guidelines. These results may support the value of targeted preventive measures and healthcare resource planning for asthma, though larger multi-year data sets are required to enhance statistical robustness.

Asthma, the most common chronic respiratory condition worldwide, affects about 300 million people globally [1]. The global burden of asthma is estimated to increase due to expanding urbanisation [2], especially in cities with high outdoor air pollution from economically driven activities [3]. Evidence indicates that air pollution has a detrimental effect on asthma outcomes in both adult and child populations [4]. The World Health Organization (WHO) identifies specific air pollutants that can trigger asthma exacerbations, including nitrogen dioxide (NO_2_) [5–7], ozone (O_3_) [6–8], sulphur dioxide (SO_2_), and fine particulate matter (PM) at 10 μm (PM_10_) and 2.5 μm (PM_2.5_) [6,7,9]. The pollutant with the most significant impact on human health is PM, which is widely used as an indicator of air quality [10]. International studies have established an association between worsening of asthma symptoms and air pollution. Gordian *et al.* in Alaska reported a 3–6% increase in hospital visits for asthma when PM_10_ concentrations rose by 10 μg/m^3^ [11]. A study in the USA found a significant association between asthma emergency visits among children and PM_10_ exposure [12]. Further evidence reported associations between exposure to carbon monoxide (CO) and NO_2_ and severe asthma in adults [13] and children [14]. A five-year study (2009–2013) in London primary care facilities found that even brief exposure to PM_10_ and NO_2_ was associated with increased visits for respiratory symptoms and inhaler prescriptions [15]. Air pollution and climate change are closely connected, forming an intertwined system that poses significant risks to human health [16]. A review reported that extreme changes in temperature or weather were associated with increased risks of asthma exacerbations, 1.25-fold (95% confidence interval (CI) = 1.14, 1.37) for emergency visits and 1.10-fold (95% CI = 1.04, 1.17) for hospital admissions [17]. Meteorological factors, such as air temperature, wind, rainfall, and humidity, can exacerbate air pollution by affecting the transport and dispersion of pollutants [18].

Malaysia has an estimated asthma prevalence of 5.3–7.1% among children and adolescents, and 6.2% among adults [19–21]. Selangor, one of the states in Klang Valley, Malaysia, has the highest prevalence of adults with asthma at 22.0% [22]. Klang Valley is densely populated with over seven million residents and numerous air-polluting industries, including factories, power plants, and transport activities [23]. The challenge of suboptimal asthma control persists, where 70% of adults continue to experience poor control [24]. An environmental trigger, haze, was reported as a main exacerbating factor in 50% of affected adults [24]. Local studies have identified associations between haze and increased hospital admissions for asthma [25] and other respiratory illnesses [26]. Another study using 2000–2009 pollution data reported that urbanisation and high vehicular emissions in Klang Valley have severely polluted the area with PM_10_, putting the population at risk of chronic respiratory problems [27]. Pollutants affect not only the respiratory system but also the cardiovascular system, with studies indicating that a 10 μg/m^3^ increase in NO_2_ concentrations was associated with a 7.32% increase in cardiovascular admissions [28].

Between 2014 and 2017, several haze episodes affected the Klang Valley. In 2014, haze occurred from February to March due to seasonal dry weather and local peatland fires. A subsequent episode from June to October 2014 was caused by transboundary haze pollution from the Southwest monsoon. Forest fires in Sumatra and Kalimantan, Indonesia, in August and September 2015, significantly worsened air quality [29]. We aimed to examine the relationships among daily pollutant concentrations, meteorological factors, and emergency visits for asthma exacerbations, using retrospective data from 2014–2017, a period that included severe haze events. We employed generalised additive models (GAMs), a flexible semi-parametric approach, to estimate the associations of emergency visits with changes in environmental factors.

## METHODS

### Study design and setting

We conducted a retrospective study in Petaling Jaya (PJ), a city in Klang Valley, where environmental data from the Department of Environment and Malaysia Meteorological Department monitoring stations, and records of emergency visits for asthma exacerbations from the Trauma and Emergency Unit of Universiti Malaya Medical Centre (UMMC) were all available for data collection (Appendix S1 in the **Online Supplementary Document**). The UMMC is a government-funded teaching hospital approximately 1.6 km from the PJ air quality station and 2 km from the PJ meteorological station. PJ is an industrial and residential urban area with high levels of road traffic congestion and pollutant emissions [30–32]. Based on patients’ addresses, 85.6% resided within 20 km of the hospital (Appendix S3 in the **Online Supplementary Document**).

### Data collection

The available data we used span from 1 January 2014 to 13 April 2017, covering both haze and non-haze periods. Several haze events occurred in 2014 and 2015, when PM_10_ levels exceeded the daily mean (x̄) limit of the Malaysian Ambient Air Quality Standard (MAAQS) 2015 of 100 μg/m^3^ [33] at multiple locations [34,35].

### Health data

We collected the health data either manually from clinical records or electronically from the Electronic Medical Records. We removed all hospital registration numbers and assigned non-identifiable identification codes before analysis. To ensure data collection was clear and systematic, we created a codebook outlining inclusion and exclusion criteria, a detailed account of the retrieved cases, and the total number of daily emergency visits. Inclusion criteria included all patients who visited the hospital’s Trauma and Emergency Unit with a documented diagnosis of asthma exacerbation by the attending physicians (referred to as ‘asthma emergency visit’ in this paper), with a documented underlying history of asthma (Appendix S2 in the **Online Supplementary Document**). This also included exacerbations secondary to upper respiratory tract infections, pneumonia, cold exposure, pets, and environmental changes. To ensure adherence to the established criteria, the lead investigator (NH) conducted an audit of a randomly selected sample. We collected age, daily attendance for asthma emergency visits, and home address data. We used the total number of patients visiting the Trauma and Emergency Unit during the study period as the denominator to calculate the asthma-related visit ratio. We focused on examining emergency visits for asthma exacerbations on a per-visit rather than per-patient basis. For patients who had repeated visits, we treated each visit as a separate incident. We considered this approach to reflect the short-term impact of air pollution on hospital workload.

### Air quality and meteorological data

We used air pollutants (*i.e.* PM_10_, CO, NO_2_, SO_2_, and O_3_) and meteorological data (*i.e.* air temperature, humidity, atmospheric pressure, wind, and rainfall) obtained from nearby stations. We calculated daily x̄ concentrations for PM_10_, CO, NO_2_, SO_2_, air temperature, relative humidity, atmospheric pressure, and wind (speed and direction) from hourly measurements. For O_3_, we used the maximum eight-hour rolling average concentration for the day, whereas we calculated daily rainfall (duration and volume) as the sum of the day. We conducted all calculations in accordance with the WHO Air Quality Guidelines (AQG) metrics [10] for assessing short-term exposure effects. We applied a data capture threshold of 75% when calculating daily data. During the study period, only PM_10_ data were available for PM, whereas PM_2.5_ data became available only after July 2017; therefore, we excluded it from the analysis.

### Data analysis

We reported age and asthma emergency visits as frequencies and percentages. We examined associations between daily visits and environmental variables using GAMs with a negative binomial distribution and log link to account for overdispersion (variance-to-x̄ ratio = 1.42) (Appendix S3 in the **Online Supplementary Document**). The model included parametric and nonlinear terms to control for covariates (*i.e.* long-term trends, seasonality, month, day of week, holidays, air pollutants, and meteorological variables). Parametric predictors included month, day, holidays, and rainfall. We modelled continuous variables (*i.e.* air pollutants and meteorological factors) with cubic splines for nonlinear relationships. We estimated smoothing parameters using restricted maximum likelihood. The model showed a good fit (Pearson dispersion = 1.006; adjusted R^2^ = 0.28; deviance = 32.1%). To explore delayed and nonlinear effects of PM_10_, we used a distributed lag nonlinear model within the GAM framework over a lag period of up to seven days, modelling nonlinear exposure-response and lag structure. We estimated relative risks (RRs) of asthma emergency visits associated with PM_10_ concentrations using 100 μg/m^3^ as the reference, corresponding to the MAAQS threshold [33].

We used *R*, version 4.5.1 (R Core Team, Vienna, Austria) for all analyses.

## RESULTS

### Emergency visits and environmental variables

A total of 10 411 asthma emergency visits were recorded from 1 January 2014 to 13 April 2017, representing 2.3% of all emergency visits during those four years (**Table 1**). The median of asthma visits per day was 8 (interquartile range = 6–11). Most patients were aged ≥18 years, with a median age of 34 years (**Table 2**). The daily x̄ PM_10_ concentration during the study period was 51.47 μg/m^3^, which was higher than the WHO AQG for the daily x̄ limit of 45 μg/m^3^ [10] but lower than the MAAQS daily x̄ limit of 100 μg/m^3^ [33] (**Table 3**).

**Table 1:** Emergency visits between 1 January 2014 and 13 April 2017

	n (%)
**Total number of all emergency visits**	450 607
**Asthma emergency visits**	10 411 (2.3)
**Daily number of asthma emergency visits, MD (IQR)**	8 (6–11)

IQR – interquartile range, MD – median

**Table 2:** Age distribution of the included participants (n = 10 411)

	n (%)
**Age in years**	
x̄ (SD)	36.3 (24.3)
MD	34
**Age group distribution in years**	
≤5	1327 (12.7)
6–11	1084 (10.4)
12–17	454 (4.4)
18–60	5239 (50.3)
>60	2292 (22.1)
Unknown	15 (0.1)

MD – median, SD – standard deviation, x̄ – mean

**Table 3:** Average values of the recorded environmental variables’ daily concentrations

	x̄ (SD)	MD (IQR)
**Air pollutants (μg/m^3^)**		
PM_10_	51.47 (31.94)	43.81 (34.41–57.06)
O_3_	99.54 (41.68)	94.82 (69.16–126.83)
NO_2_	49.05 (12.16)	48.68 (40.99–56.67)
SO_2_	11.13 (4.94)	10.65 (7.55–14.22)
CO (mg/m^3^)	1.35 (0.38)	1.31 (1.11–1.55)
**Meteorological data**		
Daily temperature (°C)	28.49 (1.28)	28.51 (27.57–29.49)
Daily relative humidity (%)	73.88 (7.69)	74.25 (68.48–79.62)
Daily wind speed (m/s)	4.90 (1.14)	1.12 (0.97–1.30)
Atmospheric pressure (hPa)	1009.00 (1.35)	1009.00 (1008.00–1010.00)
Rainfall duration (min)	82.10 (1.07)	30.00 (0.0–143.00)
Rainfall volume (mm)	9.20 (1.76)	0.20 (0.0–11.00)

IQR – interquartile range, MD – median, SD – standard deviation, x̄ – mean

### Asthma emergency visits and PM_10_

**Figure 1:**
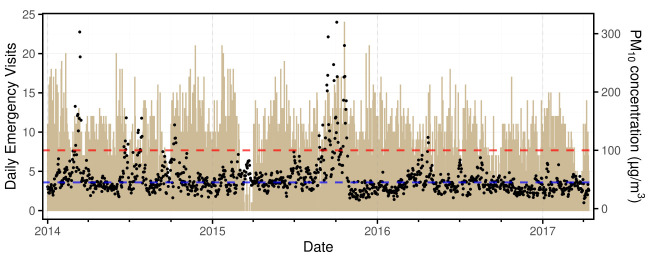
Daily count of asthma emergency visits (light brown bars) and daily mean PM_10_ concentrations (black dots) observed during the study period. The blue line indicates the World Health Organization Air Quality Guidelines for the daily mean limit of 45 μg/m^3^ [10], and the red line represents the Malaysian Ambient Air Quality Standard for the daily mean limit of 100 μg/m^3^ [33]. PM_10_ – particulate matter at 10 μm.

Peak daily asthma emergency visits occurred in early and late 2014, early 2015, and mid-to-late 2015 (**Figure 1**). This observation appeared to follow a similar pattern with the highest recorded x̄ daily PM_10_ levels of haze episodes, exceeding the daily x̄ MAAQs threshold of 100 μg/m^3^ and reaching 300–400 μg/m^3^. There were 4855 (45.6%) of asthma emergency visits when PM_10_ concentrations were above the WHO AQG thresholds, and 631 (6.06%) when they were above the MAAQS thresholds.

### Association of environmental variables with asthma emergency visits

**Figure 2:**
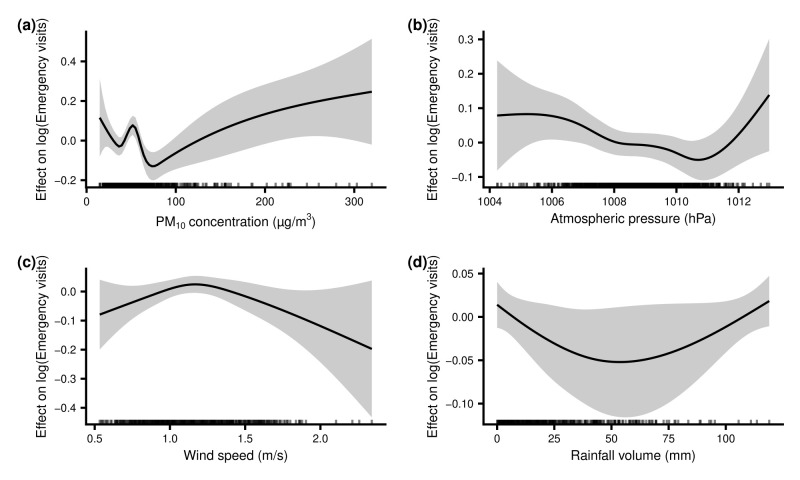
Daily mean of PM_10_ concentrations, atmospheric pressure, wind speed, and rainfall volume and their associations with daily asthma emergency visits based on GAM analysis. **Panel A.** PM_10_. **Panel B.** Atmospheric pressure. **Panel C.** Wind speed. **Panel D.** Rainfall volume. The bold black line estimates the relative effect sizes for asthma emergency visits (y-axis), and the shaded area represents the 95% confidence interval. PM10 – particulate matter at 10 μm.

PM_10_ concentration below the daily x̄ of 51 μg/m^3^ was not significantly associated with emergency visits (x̄ = −3.8%; 95% CI = −21.4, 17.8%) (**Figure 2**, Panel A). With a PM_10_ increase from 51 μg/m^3^ to 100 μg/m^3^, there was a 12.8% (95% CI = −20.8, −4.1) reduction in emergency visits. The association became positive at higher concentrations. A PM_10_ increase from 100 to 200 μg/m^3^ was associated with a 21.3% (95% CI = 2.2, 44.0) increase, while an increase from 100 to 300 μg/m^3^ corresponded to a 34.0% (95% CI = 4.7, 71.7) increase in emergency visits. The PM_10_ increase from 200 to 300 μg/m^3^ was associated with an emergency visit increase of 10.5% (95% CI = −16.3, 45.8) but was not statistically significant, most likely due to fewer observations (**Figure 1**, **Figure 2**, Panels A). Overall, the results indicate a threshold effect, in which the association between PM_10_ and asthma emergency visits becomes apparent only above the MAAQS daily x̄ limit of 100 μg/m^3^. The relationship with atmospheric pressure was nonlinear (estimated degrees of freedom (EDF) = 2.98; *P* = 0.01) (**Figure 2**, Panel B), whereas the relationship with wind speed was moderately nonlinear (estimated degrees of freedom = 2.2, *P* = 0.01) (**Figure 2**, Panel C). Across the central 90% of the observed atmospheric pressure range (1007–1011 hPa), a 4.35 hPa increase was associated with a 12.8% (95% CI = −17.7, −2.3) reduction in emergency visits. An increase in wind speed from 1 to 2 m/s was associated with a 12.0% (95% CI = −22.5, −0.1) decrease in emergency visits. For rainfall (**Figure 2**, Panel D), an increase in volume from 0 to 50 mm was associated with a decrease in emergency visits by 6.4% (95% CI = −12.5, 0.2). The increase in rainfall from 50 to 100 mm was associated with an increase in emergency visits of 4.11% (95% CI = −2.9, 11.5); however, it was not statistically significant.

### Patterns of asthma emergency visits during the study period

We analysed patterns of asthma emergency visits across days of the week, revealing statistically significant variations. Compared with the Friday baseline, Mondays (21.8%; *P* < 0.001), Tuesdays (13.3%; *P* = 0.001), and Sundays (9.5%; *P* = 0.02) showed increases in asthma visits. The public holiday effects were not statistically significant, with no evident seasonal trend observed.

### The lag effect

**Figure 3:**
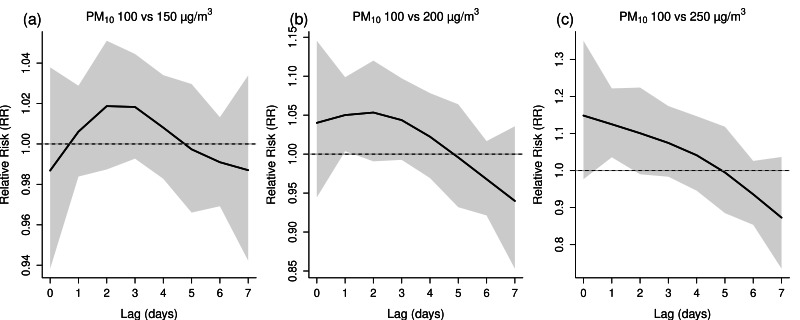
The RR of asthma emergency visits at various daily mean PM_10_ concentrations compared to the daily MAAQS threshold. **Panel A.** PM_10_ 100 *vs.* 150μg/m^3^. **Panel B**. PM_10_ 100 *vs.* 200μg/m^3^. **Panel C.** PM_10_ 100 *vs.* 250μg/m^3^. The bold line indicates the point estimate, and the horizontal line at relative risk = 1 denotes no excess risk, while values >1 represent increased risks. The shaded area is 95% confidence interval. PM_10_ – particulate matter at 10 μm, RR – relative risk.

The distributed lag nonlinear model analysis showed that at a PM_10_ concentration of 150 μg/m^3^, the RR ranged from 0.995 to 1.015, with the highest lag effects observed on days two and three (**Figure 3**, Panel A). When the concentration increased to 200 μg/m^3^, there was a 5.3% increase in emergency visits on the same day (lag 0) and persisted through day two (**Figure 3**, Panel B). At 250 μg/m^3^, the RR was 1.165 on day 0, indicating a 16.5% increase in emergency visits on the same day (**Figure 3**, Panel C).

### Cumulative exposure-response relationship

The cumulative exposure-response curve (lag 0–7) illustrates a positive, nonlinear relationship between PM_10_ concentrations and RR of asthma emergency visits (Appendix S4 in the **Online Supplementary Document**). The risk remains relatively stable at 100 μg/m^3^ (MAAQS threshold) but begins to show a noticeable upward trajectory as levels exceed 150 μg/m^3^, reaching RR values above 1.5 at the highest observed concentrations. However, the differences were not statistically significant. We used the lag window of 0–7-day in the model to demonstrate the short-term effects of PM_10_ exposure. We have examined the cross-correlation between PM_10_ and asthma emergency visits over lags from −15 to 15 days and found significant lags within 0–7 days (Appendix S5 in the **Online Supplementary Document**).

## DISCUSSION

We found that from 1 January 2014 to 13 April 2017, asthma exacerbations accounted for 2.3% of all emergency visits at UMMC. The highest number of visits occurred in early 2014 and mid-to-late 2015. This appeared to follow a similar pattern with the highest recorded x̄ daily PM_10_ levels observed in Klang Valley. Higher concentrations of PM_10_ above the MAAQS threshold were associated with increased risk of asthma emergency visits. Lower atmospheric pressure and wind speed were significantly associated with increased risk, while moderate rainfall volume was associated with reduced risk of emergency visits. The lag effect on asthma emergency visits was influenced by PM_10_ exposure level, with the risk peaking on days two and three at 100 μg/m^3^ and on the same day at 200 μg/m^3^ and 250 μg/m^3^. The cumulative exposure-response curve from lag 0–7 days shows that the RR of asthma emergency visits began at PM_10_ levels of 150 μg/m^3^. There was a significant increase in asthma visits on Mondays (21.8%; *P* < 0.001), Tuesdays (13.3%; *P* = 0.001), and Sundays (9.5%; *P* = 0.02).

Our findings on the association between PM_10_ concentrations and the risk of asthma emergency visits were consistent with several other studies [3,36–38]. We observed that the association was nonlinear. At 100 μg/m^3^, there was a minimal or decreased risk of emergency visits. However, when levels exceeded the MAAQS threshold of 100 μg/m^3^, there was an increase in the number of visits of 21.3% at 200 μg/m^3^ and of 34.0% at 300 μg/m^3^. Although a positive trend is suggested, the model lacks the statistical power to confirm a significant effect at concentrations above 200 μg/m^3^ and should be interpreted with caution. Nevertheless, the associated increase in emergency visits may represent a clinically meaningful trend and may justify proactive measures for patients with asthma and healthcare facilities.

Regarding meteorological factors, other studies have reported inconsistent findings on the relationship between wind speed and asthma exacerbations. Some studies have indicated that higher wind speeds are associated with increased asthma emergency visits [39,40]. The explanation was that high winds can disperse PM, pollen, and mould spores, increasing their presence in the air and making them more likely to be inhaled by people with asthma [39]. In contrast, we found higher wind speeds to be associated with decreased asthma emergency visits. A possible explanation is that PM_10_ is readily transported over long distances by wind [41]. Low wind speeds can reduce pollutant dispersion, leading to higher pollutant levels in the area [42] and potentially triggering asthma exacerbations. These inconsistencies in results could arise from variations in cohorts, age groups, settings, or geographical locations, and future studies are needed to confirm this. Regarding atmospheric pressure, our findings were similar to those from Beijing, reporting a negative correlation with daily asthma hospital admissions [43]. While the precise physiological mechanisms by which atmospheric pressure influences asthma are not fully understood, it could be hypothesised that lower pressure often coincides with extreme weather changes, such as thunderstorms, which can trigger asthma symptoms [44]. The association between rainfall volume and asthma has also been controversial; however, a moderate amount has been reported to have a protective effect, consistent with our findings, which is attributed to its removal of particulates and allergens from the atmosphere [45].

Asthma emergency visit patterns also indicated that weekdays accounted for most daily visits, specifically Monday, followed by Tuesday and Sunday. In PJ, Sunday is a designated public holiday, with Monday and Tuesday following. The surge in emergency visits on these days is likely due to primary or outpatient clinics being closed or to a backlog of cases accumulated over the weekend, when clinics are closed. Additionally, this pattern may also be influenced by the delayed physiological response to air pollution exposure, since symptoms can take a few days to worsen enough to require emergency care. These findings may also be related to increased human mobility and activity on weekends, leading to greater exposure to environmental pollutants.

The lag effect of PM_10_ concentrations on the risks of asthma emergency visits varies. At 150 μg/m^3^, which is above the MAAQS threshold, the peak effect occurred on days two and three. When PM_10_ exceeded 200 μg/m^3^, a higher risk was observed on the same day (lag 0) and persisted up to three days. The lag effect observed on the same day at higher concentrations may have been due to higher PM_10_ levels, which could have prompted vulnerable individuals to visit the hospital earlier, leading to fewer visits on subsequent days. This is known as a harvesting effect or short-term displacement: people seek care earlier due to earlier exposure to high concentrations, thereby decreasing the number of people seeking care in the following days [46]. Although it may appear speculative, from a public health perspective, it suggests that patients might remain indoors or take preventive medication during periods of moderate to high pollution, resulting in lower emergency visits. The harvesting effect, where elevated pollution levels at 200 μg/m^3^ and 300 μg/m^3^ accelerate health events that would have occurred shortly afterwards, could account for the increased emergency visits observed on the same day. It is important to note that asthma exacerbations can still occur up to 2–3 days after exposure. The observed pattern of lagged effects over several days post-exposure to PM_10_ appears biologically plausible, as inflammatory responses to air pollution may require several days to manifest as symptoms severe enough to necessitate hospital visits [47]. This insight can assist public health authorities in issuing warnings that cover a longer period of vulnerability following a pollution event. Such warnings might encourage people to stay indoors or prompt authorities to take actions like closing schools, which could subsequently reduce exposure. The cumulative exposure-response curve from lag 0–7 days shows that the increased risk of asthma emergency visits began at PM_10_ levels of 150 μg/m^3^. It remains unclear whether the daily x̄ PM_10_ threshold of 100 μg/m^3^, set by the MAAQS, is ideal or appropriate given this narrow margin. However, these findings are preliminary because the wide CI limits definitive conclusions. Further research using larger, multi-year data sets is needed to narrow CIs and strengthen the findings, which could help shape regional air quality policies and clinical planning in the Klang Valley.

This study has several limitations. We obtained the health data from a single main government hospital, which could limit the generalisability of the findings to the broader population, as it could not have detected all emergency visits within the Klang Valley. We initially considered gathering data from multiple public/private hospitals and clinics, but the lack of systematic record-keeping meant that collecting accurate retrospective daily clinical records was not possible. We selected the most reliable data source available for our time-series analysis, but acknowledge that the risk estimates for emergency visits associated with PM_10_ levels was limited to a single catchment area and should be interpreted with caution. Second, the retrospective nature of this study imposes certain limitations. While we used the x̄ daily concentration to estimate population exposure, it may not accurately reflect individual exposure levels due to unaccounted confounding factors, such as indoor air pollution, smoking, socioeconomic status, and pre-existing co-morbidities. These limitations could introduce bias in assessing the effects of air pollution, and therefore, the findings should be interpreted with caution. Further prospective studies are needed to strengthen the findings. Third, regarding data recording practices, we acknowledge that not using International Classification of Diseases coding may lead to variability in data entry. This also represents a limitation in retrospective studies. Additionally, concerning time-varying confounding, we incorporated only day of the week, month of the year, and public holidays within the GAM model (Appendix S3 in the **Online Supplementary Document**). We omitted other potential variables, such as infectious respiratory epidemics and school holidays, which could potentially introduce bias into the exposure-response relationship. Future prospective studies should consider these variables to ensure that the estimated PM_10_ risks are independent of temporal variations. Additionally, we made assumptions that all asthma exacerbations would present to the UMMC emergency unit, which might not be true, suggesting that the true impact of pollution could be underestimated. Our study also has several strengths. We chose emergency visits for asthma exacerbations as the outcome variable rather than hospital admissions, which better captures the possible association with pollution. GAMs, the modelling technique used in this study, have provided a strong framework for data analysis, helping to address the potential nonlinearity of air pollution exposure and better understand the association between pollution and health service use.

## CONCLUSIONS

Although we used data from a single facility and may not be fully representative of the entire Klang Valley population, we provide an important 'sentinel' perspective, potentially contributing to a broader understanding of environmental triggers in the Klang Valley. Our findings suggest that PM_10_ exposure levels above MAAQS thresholds may coincide with a period of increased risk for patients with asthma, highlighting the potential value of targeted preventive measures. Given the observed association of pollution with emergency care utilisation, further consideration of the alignment between local thresholds and international WHO guidelines may help better protect vulnerable populations and inform health care resource planning. The observed narrow margin of PM_10_ concentrations between 100 and 150 μg/m^3^ (MAAQS specifies a limit of 100 μg/m^3^) offers preliminary insights. However, these results should be regarded as exploratory, necessitating further investigation through analysis of larger, multi-year data sets to narrow CIs and enhance the robustness of the findings to inform regional air quality policies and clinical preparedness in the Klang Valley.

## Additional material


Online Supplementary Document
