# Supplementary material for: Time series analysis of environmental factors and their association with emergency visits for asthma exacerbations in Petaling Jaya, Malaysia
Source: J Glob Health. 2026 Aug 7;16:04249. doi: 10.7189/jogh.16.04249 (PMC13449562; doi:10.7189/jogh.16.04249)
Supplement: Supplementary file 1 [file jogh-16-04249-s001.pdf]

Supplement to: Hussein N, Cheah W, Lin C, Fong CS, Aghamohammadi N, Hanafi NS, Pang YK, Yahya MS, Nor ATM, Pinnock H, Campbell H, Khoo EM. Time series analysis of environmental factors and their association with emergency visits for asthma exacerbations in Petaling Jaya, Malaysia. J Glob Health. 2026;16:04249.

Appendix S1. A Map of the Klang Valley with data source landmarks in Petaling Jaya

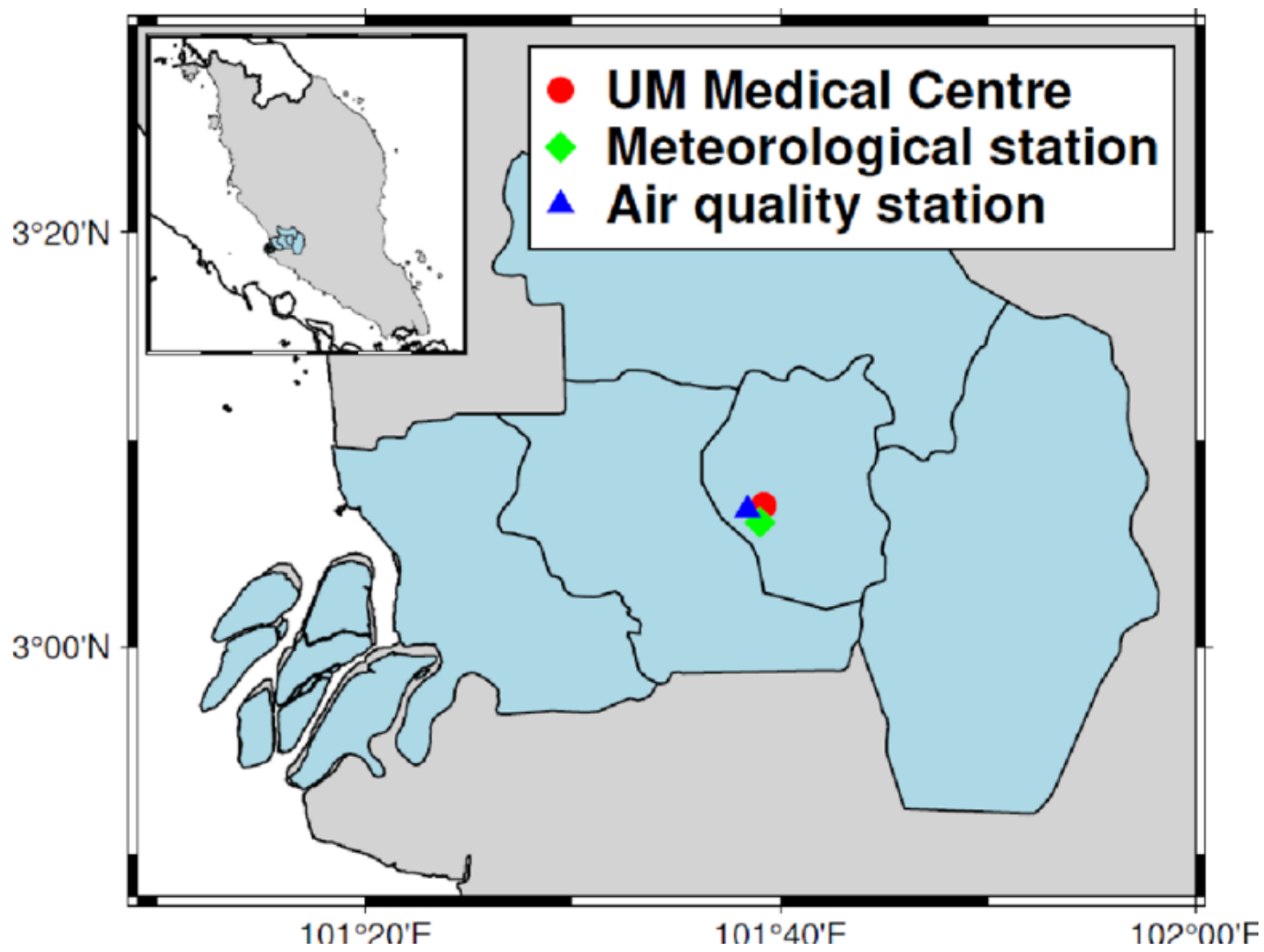

The hospital UMMC is a government-funded teaching hospital approximately 1.6 kilometers from the PJ air quality station (Station No. 44304; 2.983°N, 101.483°E) and 2 kilometers from the PJ weather station (Station No. 48648; 3.100°N, 101.650°E)

Appendix S2. Inclusion and exclusion criteria

The diagnoses were extracted from patients' clinic records.

Inclusion criteria were all patients who visited the hospital’s Trauma and Emergency Unit with a documented diagnosis of asthma exacerbation by the attending physician (referred to as an ‘asthma emergency visit’ in this paper) and a documented history of asthma.

And/Or secondary to documented:

Upper respiratory tract infections, pneumonia, cold exposure, pet, environmental changes.

Diagnoses below with no documented asthma in the records were excluded:

- Acute bronchiolitis
- Acute bronchitis
- Acute pharyngitis
- Acute tonsillitis
- Bronchiectasis
- Bronchiectasis
- Bronchopneumonia
- Bronchospasm
- Chronic obstructive airway disease
- Community-acquired pneumonia
- Coryza
- Hospital-acquired pneumonia
- Hyperactive airway disease
- Pertussis
- Pneumonia
- Upper respiratory tract infection
- Viral croup
- Viral-induced wheeze

Appendix S3. Methods

Patient Address Distance to UMMC

Patient addresses were geocoded using the Nominatim OpenStreetMap geocoder via the tidygeocoder package in R. Because address quality varied, five-digit Malaysian postcodes were extracted and used for geocoding. Straight-line distances from each patient's location to UMMC were calculated using the Haversine formula with the geosphere package. Records with valid distance estimates, 85.6% was within 20 km of UMMC.

Calculation of Air Quality data

The 8-hour rolling average concentration at a particular hour was calculated as the mean of the hourly concentrations for that hour and the preceding seven hours. This moving average was computed hourly, advancing one hour at a time, resulting in up to 17 valid 8-hour averages per day, assuming complete data. A data capture threshold of 75% was applied when calculating daily pollutant averages. For O_3, any 8-hour rolling average was considered missing if it included fewer than six hourly concentrations. In addition, if there were fewer than 18 valid 8-hour rolling averages available in a day, the daily maximum 8-hour average for O_3 was also classified as missing. For other pollutants, the daily mean concentration was considered missing if fewer than 18 hourly values were recorded in a 24-hour period.

Calculation of Meteorological data

Days with fewer than 10 hourly data points were excluded and treated as missing data. A total of 98 missing values were identified for temperature, relative humidity and atmospheric pressure. These gaps were imputed using linear regression models.

Statistical Analysis Methods

Preliminary Data Assessment

Prior to constructing the predictive models, the distribution and relationships within the dataset were evaluated. The normality of continuous environmental variables (e.g., rain volume) was assessed using the Shapiro-Wilk test. To determine the appropriate probability distribution for the daily count of asthma visits, we calculated the variance-to-mean ratio. The calculated dispersion ratio was 1.52, which exceeds the standard threshold of 1.0 for a Poisson distribution. Consequently, a standard Poisson model was deemed inappropriate due to overdispersion, and a negative binomial distribution was selected to model the count data. Additionally, we assessed potential multicollinearity by calculating the correlation matrix for all continuous air pollutants (PM_{10}, CO, NO_2, NO, NO_x, SO_2, and O_3) to ensure highly correlated variables did not confound the estimates.

Generalised Additive Model (GAM)

A generalised additive model (GAM) with a negative binomial distribution was fitted to examine the associations between daily asthma visits, air pollutants, and meteorological factors. Smoothing parameters were estimated using restricted maximum likelihood (REML). To aid variable selection and minimize overfitting, an additional penalty was imposed on the smoothing basis (select = TRUE), allowing non-informative nonlinear terms to be penalised to zero. Alternative model specifications were compared using the Akaike Information Criterion (AIC), and the model with the lowest AIC was retained. All parameters were retained in the final model.The final model adjusted for temporal and categorical confounders. Long-term trend and seasonality were controlled using a cubic regression spline (bs = "cr", k = 250) for the time variable. Day of the week, month of the year, public holidays, and a binary rainfall indicator were included as linear parametric terms. Environmental exposures were modelled using penalised splines to allow for nonlinear associations. Cubic regression splines were used for PM_{10}, SO_2, and atmospheric pressure (k = 10), and for O_3, CO, NO_2, wind speed, and rain volume (k = 5), while thin plate regression splines were used for temperature and relative humidity.

Model Diagnostics

Model adequacy was evaluated using standard GAM diagnostics (gam.check) to ensure that the selected basis dimensions were sufficient and that the smooth terms were not oversmoothed. Residual overdispersion was assessed using the Pearson dispersion statistic, with values close to 1.0 indicating adequate fit of the negative binomial model.

As associations were modelled nonlinearly, effects were quantified by comparing predicted log-risk values between selected exposure levels. For each contrast, the difference in the estimated smooth function was calculated and exponentiated to obtain the relative risk, which was then expressed as a percentage change in daily asthma visits. Ninety-five percent confidence intervals were derived from the standard error of the difference. This approach was applied to selected contrasts for PM_{10}, atmospheric pressure, rainfall volume, and wind speed.

Distributed Lag Non-linear Modelling (DLNM)

Delayed effects of PM_{10} on asthma visits were assessed using a distributed lag non-linear model (DLNM) fitted with the dlnm package in R. A cross-basis function was specified for PM_{10} across lags 0-7 days using natural cubic splines with 4 degrees of freedom for both exposure and lag dimensions. This term was incorporated into the main GAM with the same covariate adjustments as the primary model. Predicted cumulative and lag-specific associations were estimated using crosspred function for PM_{10} concentrations from 0 to 300

μg/m^3 in 5-unit increments, centred at the sample mean (51.4 μg/m^3), the WHO 24-hour guideline (45.0 μg/m^3), and the Malaysian standard (100.0 μg/m^3).

Appendix S4. Cumulative exposure-response relationship between daily mean of PM_{10} concentrations and relative risk (RR) asthma emergency visits over lag 0–7 days

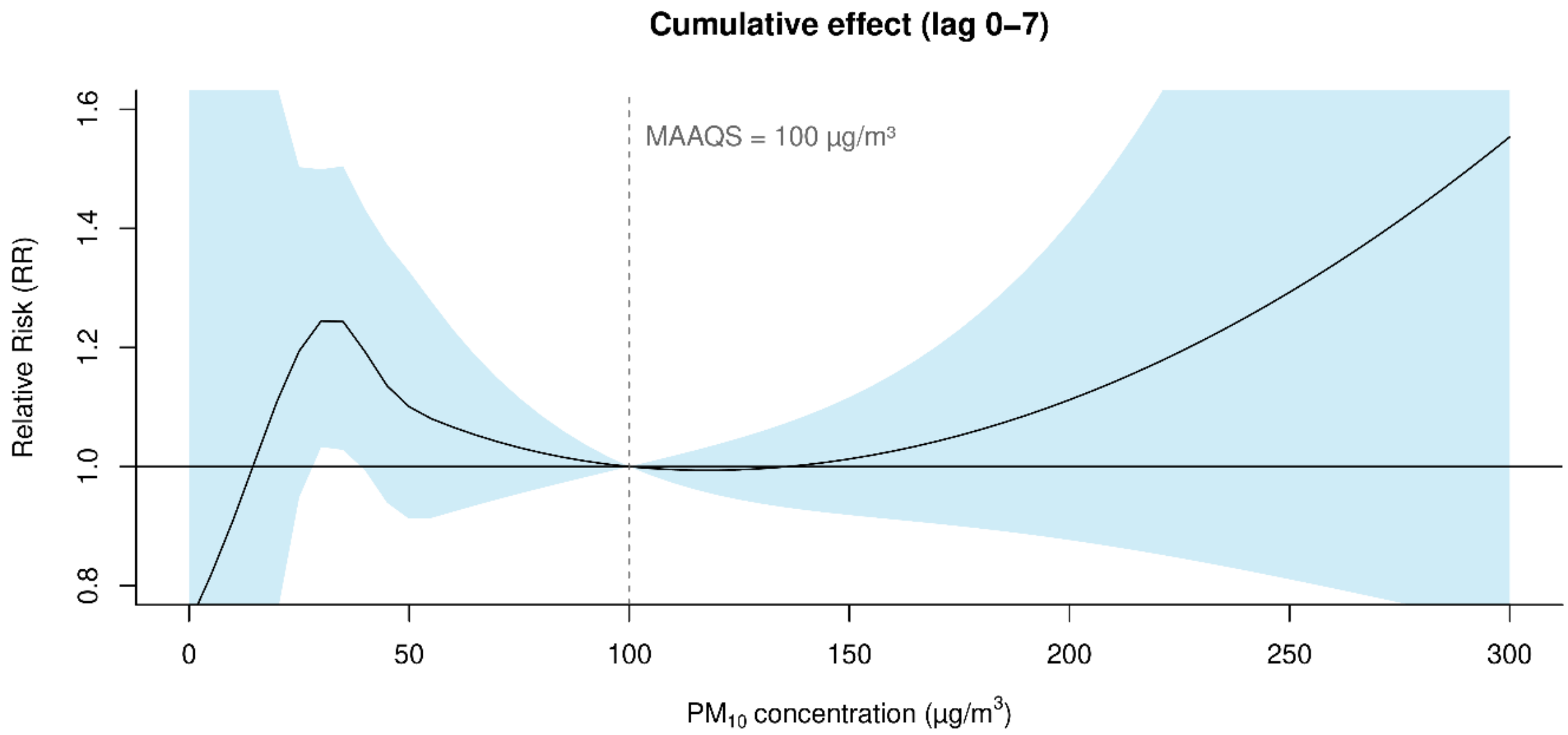

*The relative risk (RR) of asthma emergency visits and 95% confidence intervals are shown for PM_{10} concentrations (centred at the MAAQS daily mean limit of 100 µg/m³). The shaded area is 95% CI.

Appendix S5. Cross-correlation between PM_{10} and asthma emergency visits over lags from −15 to 15 days

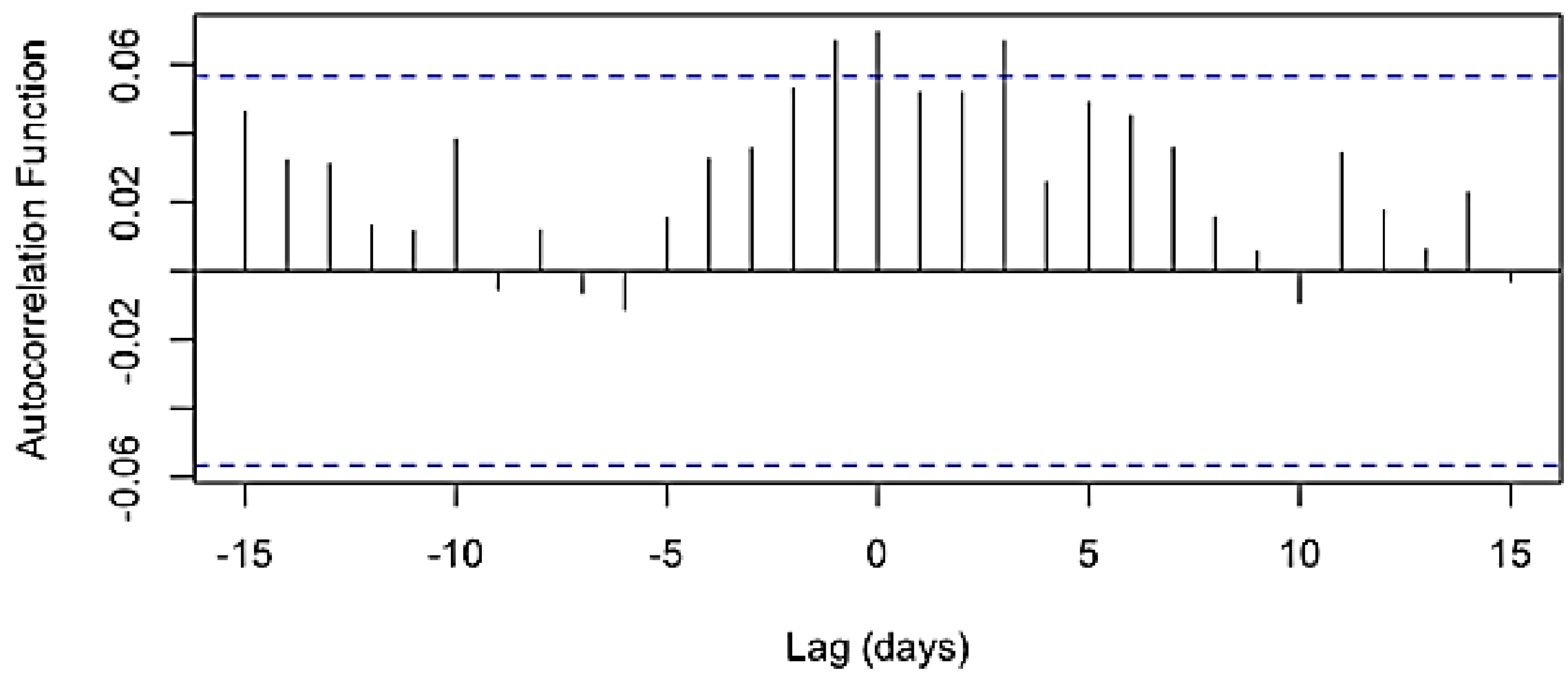

The plot shows the auto-correlation function (ACF) between daily mean PM_{10} concentrations and daily asthma emergency visits across different lag periods, from -15 to +15 days. The blue dashed lines represent the significance threshold.
